# TBI lung dose comparisons using bilateral and anteroposterior delivery techniques and tissue density corrections

**DOI:** 10.1120/jacmp.v16i2.5293

**Published:** 2015-03-08

**Authors:** Daniel W. Bailey, Iris Z. Wang, Tara Lakeman, Lee D. Hales, Anurag K. Singh, Matthew B. Podgorsak

**Affiliations:** ^1^ Department of Radiation Oncology Northside Hospital Cancer Institute Atlanta GA; ^2^ Department of Radiation Medicine Roswell Park Cancer Institute Buffalo NY; ^3^ Department of Physiology and Biophysics State University of New York at Buffalo Buffalo NY USA

**Keywords:** radiation therapy, total body irradiation, TBI, dosimetry, lung dose, heterogeneity correction, tissue density correction, inhomogeneity corrections

## Abstract

This study compares lung dose distributions for two common techniques of total body photon irradiation (TBI) at extended source‐to‐surface distance calculated with, and without, tissue density correction (TDC). Lung dose correction factors as a function of lateral thorax separation are approximated for bilateral opposed TBI (supine), similar to those published for anteroposterior–posteroanterior (AP–PA) techniques in AAPM Report 17 (i.e., Task Group 29). 3D treatment plans were created retrospectively for 24 patients treated with bilateral TBI, and for whom CT data had been acquired from the head to the lower leg. These plans included bilateral opposed and AP–PA techniques—each with and without — TDC, using source‐to‐axis distance of 377 cm and largest possible field size. On average, bilateral TBI requires 40% more monitor units than AP–PA TBI due to increased separation (26% more for 23 MV). Calculation of midline thorax dose without TDC leads to dose underestimation of 17% on average (standard deviation, 4%) for bilateral 6 MV TBI, and 11% on average (standard deviation, 3%) for 23 MV. Lung dose correction factors (CF) are calculated as the ratio of midlung dose (with TDC) to midline thorax dose (without TDC). Bilateral CF generally increases with patient separation, though with high variability due to individual uniqueness of anatomy. Bilateral CF are 5% (standard deviation, 4%) higher than the same corrections calculated for AP–PA TBI in the 6 MV case, and 4% higher (standard deviation, 2%) for 23 MV. The maximum lung dose is much higher with bilateral TBI (up to 40% higher than prescribed, depending on patient anatomy) due to the absence of arm tissue blocking the anterior chest. Dose calculations for bilateral TBI without TDC are incorrect by up to 24% in the thorax for 6 MV and up to 16% for 23 MV. Bilateral lung CF may be calculated as 1.05 times the values published in Table 6 of AAPM Report 17, though a larger patient pool is necessary to better quantify this trend. Bolus or customized shielding will reduce lung maximum dose in the anterior thorax.

PACS numbers: 87.55.D, 87.55.Dk, 87.55.Ne, 87.56.Bd, 87.57.Qp

## I. INTRODUCTION

Radiation pneumonitis is one of the most serious complications associated with total‐ and half‐body irradiation, resulting in fatality in up to 80% of presenting patients who have received uniform or near‐uniform dose to the total lung.^(1)^ Consequently, an accurate analysis of whole‐lung dose is essential in any large‐field thorax irradiation setting. In this study, lung dose distributions are compared for two common delivery techniques of total body photon irradiation (TBI). Bilateral‐opposed^2^, ^3^, ^4^, ^5^ and anteroposterior‐opposed^2^, ^6^, ^7^ (AP–PA) techniques were planned via 3D treatment planning system at extended source‐to‐axis distance both with, and without, tissue density correction (TDC), for 24 patients. The goal of this study is two‐fold: to examine the differences between lung doses calculated with inhomogeneity corrections and without these corrections (as is commonly performed in simple TBI calculations); and to compare lung dose distributions between the bilateral and AP–PA techniques. Ultimately, we aim to approximate lung dose correction factors for bilateral TBI simple calculations (i.e., non‐3D, no tissue density correction) based solely on the index of bilateral thorax separation, similar to the correction factors reported previously for the AP–PA technique by Van Dyk et al.^2^, ^6^

## II. MATERIALS AND METHODS

Twenty‐four patients for whom 3D CT data had previously been acquired from the head to the midthigh were selected for this study, representing a large range of lateral thorax separations (28–63 cm, including arms). For each patient, four treatment plans were created within the Varian ECLIPSE treatment planning platform (version 10 with AAA 10.0.28; Varian Medical Systems, Palo Alto, CA) at extended SAD of 377 cm and with $40\times 40\;{\text{cm}}^{2}$ field size: 1) bilateral opposed with TDC, and 2) without TDC, 3) AP–PA with, TDC and 4) without TDC. In each plan, dose was prescribed to a single point: the patient midline (i.e., intersection of midline sagittal and coronal planes) at the level of the umbilicus. All dose analysis was performed relative to the prescription dose, such that the results pertain to any dose prescription — however, since a defined dose prescription is necessary for calculation purposes, we used 200 cGy in 1 fraction. Extended SSD dosimetry measurements using ion chamber in water tank and radiochromic film in phantom verified the accuracy of the dose calculation and beam model at extended SSD, confirming the findings of Hussain et al.^(8)^

The plans calculated without TDC were intended to mimic the simple monitor unit (MU) calculation that would conventionally be completed for extended‐distance TBI. For each technique (i.e., bilateral and AP‐PA), the ${\text{TDC}}_{\text{off}}$ MU setting was subsequently used as a preset value in calculating the ${\text{TDC}}_{\text{on}}$ plan, yielding the dose distributions that actually occur in the inhomogeneous body from an output setting calculated with assumed homogeneity.

Values recorded for comparison included MU per field for ${\text{TDC}}_{\text{off}}$ calculation, and the following dose indices (each with and without TDC): midline thorax dose, midlung point dose for each lung, and mean and maximum (i.e., to 1 cc) lung dose. These methods were performed for the same pool of patients with both 6 MV and 23 MV photon beams.

## III. RESULTS & DISCUSSION

### A. 6 MV TBI

In terms of dose to the umbilicus (i.e., the prescription point), both AP–PA and bilateral TBI techniques calculated without inhomogeneity corrections were capable of delivering dose to the prescription point with good accuracy. For the bilateral technique, using the monitor units calculated with ${\text{TDC}}_{\text{off}}$ and recalculating with applied inhomogeneity corrections, dose delivered to the midline umbilicus was 99.3% of the prescribed dose (on average) with a standard deviation of 2.1%. The same analysis with the AP–PA technique yields a dose delivered to the midline umbilicus of 99.4% (average) and standard deviation of 1.1%. According to this analysis, inhomogeneity corrections do not have a tremendous impact on doses calculated at the umbilicus level. However, tissue density corrections have a much larger impact on planned dose distributions in the thorax. Table 1 displays the results of several indices for all patients, related to general dose delivery and midline thorax dose, and listed in order of increasing lateral thorax separation. Table 2 displays similar results but focuses on indices pertaining to lung dose. These data indicate several results of note.

Since patient separation at the umbilicus is larger in the lateral dimension than in the anteroposterior dimension, the MU required to deliver the same dose to the midline umbilicus is always higher for bilateral TBI as compared to the AP–PA technique. Our calculations indicate that bilateral TBI requires an average of 40% higher MU, ranging from approximately 20% higher than AP–PA TBI for bilateral separation of 28 cm to greater than 50% higher for larger patients. Secondly, due to the greater tissue inhomogeneity along the beam path in bilateral TBI, calculated dose at the midline thorax is greatly affected by tissue density corrections. Column four of Table 1 shows that midline thorax dose is underestimated by an average of 17% when calculations are completed without consideration of tissue inhomogeneities. This is not the case for AP–PA TBI, since the beams do not pass through substantial variation in tissue density, and thus tissue density corrections have negligible effect on midline thorax dose.

In Table 2, displayed results are related to lung dose and again sorted by increasing thorax separation. Columns three and four display the ratios of bilateral/AP–PA maximum and mean lung dose, respectively, resulting from plans calculated with TDC_on_. Notice that the mean lung dose resulting from bilateral TBI is very close to that from the AP–PA technique. However, the maximum lung dose may be significantly higher in bilateral TBI: for our patient sample, an average of 12% hotter than the maximum AP–PA lung dose. These results are due to the anatomy of the thorax, typically extending anterior to the arms (when held adjacent to the patient's sides) with separation tapering significantly toward the extreme anterior thorax. Behind the arms, the lungs experience significant dose shielding in bilateral TBI. Consequently, the maximum lung dose always occurs anterior to the arms, and in the portion of the thorax with least separation. For this reason, it may be preferable to use bolus stacked on top of the arms (e.g., saline bags) in order to even out the separation of the anterior thorax and reduce dose in this portion of the lungs.

**Table 1:** 6 MV TBI midline thorax dose results showing 6 MV bilateral vs. AP–PA TBI results for midline thorax dose, listed in order of increasing bilateral thorax separation

*# of Patients*	*Bilat. Thorax Separation (cm)*	*Bilat./AP MU Ratio*	*Bilat. Thorax Dose* $TDC_{on}/TDC_{off}$ *Ratio*	*AP Thorax Dose* $TDC_{on}/TDC_{off}$ *Ratio*
2	28	1.21	1.08	1.00
1	32	1.10	1.20	1.01
1	40	1.45	1.11	1.00
2	41	1.38	1.18	0.99
3	42	1.35	1.18	0.98
2	43	1.41	1.21	0.99
1	44	1.38	1.21	0.99
2	46	1.47	1.24	0.99
1	47	1.54	1.18	0.98
2	48	1.41	1.17	0.99
3	49	1.47	1.15	0.99
1	52	1.46	1.16	0.99
1	53	1.53	1.18	0.98
1	58	1.40	1.15	0.99
1	63	1.49	1.11	0.99
Mean		1.40	1.17	0.99
SD		0.12	0.04	0.01

**Table 2:** 6 MV TBI lung dose results showing 6 MV bilateral vs. AP–PA TBI results for lung dose, listed in order of increasing bilateral thorax separation. All ratios calculated from plans with TDC on, except the lung dose correction factors (CF). CF values represent the ratio of mean lung dose from fixed MU calculated with tissue density correction divided by the same lung dose parameter calculated without tissue density correction. Ratio of bilateral and AP–PA correction factors (column 6) is calculated for mean lung dose

*# of Patients*	*Bilat. Thorax Separation (cm)*	*Max Lung Dose Bilat./AP Ratio*	*Mean Lung Dose Bilat./AP Ratio*	*Mean Lung Dose Bilat. CF*	*Bilat./AP CF Ratio*
2	28	1.08	1.00	1.08	1.08
1	32	1.04	0.93	1.07	1.00
1	40	1.10	1.01	1.11	1.06
2	41	1.19	1.05	1.13	1.07
3	42	1.06	0.96	1.13	1.10
2	43	1.21	1.04	1.17	1.09
1	44	1.13	0.97	1.05	0.99
2	46	1.18	0.97	1.15	1.09
1	47	1.28	1.04	1.07	1.01
2	48	1.07	0.92	1.12	1.09
3	49	1.09	0.94	1.07	1.01
1	52	1.07	0.97	1.13	1.06
1	53	1.06	0.87	1.10	1.04
1	58	1.13	0.87	1.14	1.08
1	63	1.05	0.87	1.10	1.02
Mean		1.12	0.96	1.11	1.05
SD		0.07	0.06	0.03	0.04

As mentioned in the Materials & Methods section, a main purpose for this work was to examine lung doses resulting from typical simple TBI MU calculations (i.e., without tissue density corrections or 3D planning), but analyzed via a treatment planning system that accounts for tissue inhomogeneities. Consequently, the ${\text{TDC}}_{\text{off}}$ plans were used to determine the required MU as approximated for a unit‐density patient. These MU settings were then used as preset values when calculating the ${\text{TDC}}_{\text{on}}$ plans, effectively demonstrating what is really happening in the lung tissue when output is calculated with a traditional TBI calculation. The term “correction factor” (CF) in Table 2 refers to the ratio of lung dose calculated with tissue density correction divided by the same lung dose parameter calculated without tissue density correction. These correction factors allow approximation of TBI lung dose based on the independent parameter of thorax separation, analogous to the work of Van Dyk et al.^2^, ^6^ discussed on pages 29–30 (and culminating in Table 6) of AAPM Task Group 29.

**Figure 1:**
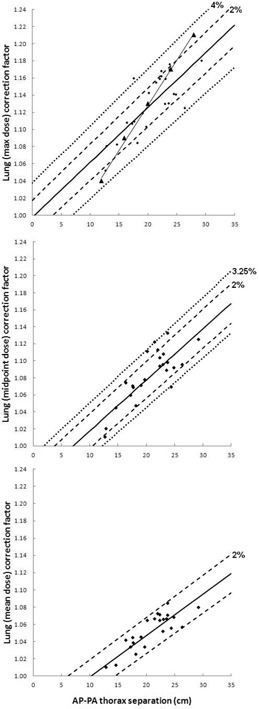
6 MV AP–PA TBI lung dose correction factors. 6 MV AP‐PA thorax separation vs. lung correction factor, with linear fit, as calculated using lung maximum dose (top), lung midpoint dose (middle), and lung mean dose (bottom) for all 24 patients. Dashed trendlines indicate ±2% from the linear fit, as well as the percent error (i.e., ±%) for which 95% of the data points are included. Allowing x to represent AP–PA separation and y to represent lung dose correction factor, the linear trendlines are described as follows: (top) y=0.0064x+0.9977, (middle) y=0.006x+0.9576, and (bottom) y=0.0048x+0.951. Triangular markers (with line for visualization purposes) indicate correction factors for 6 MV from Table 6 in TG‐29.^(2)^

**Figure 2:**
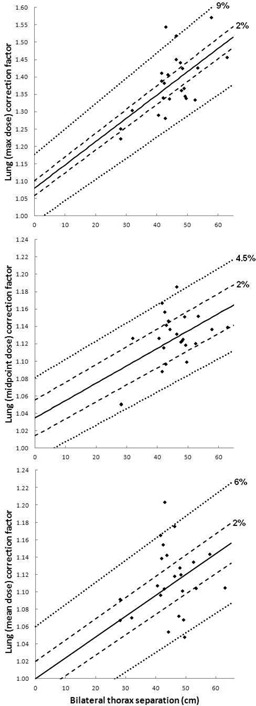
6 MV bilateral TBI lung dose correction factors. 6 MV bilateral thorax separation vs. lung correction factor, with linear fit, as calculated using lung maximum dose (top), lung midpoint dose (middle), and lung mean dose (bottom) for all 24 patients. Dashed trendlines indicate ±2% from the linear fit, as well as the percent error (i.e., ±%) for which 95% of the data points are included. Allowing x to represent bilateral separation and y to represent lung dose correction factor, the linear trendlines are described as follows: (top) y=0.0067x+1.0799, (middle) y=0.002x+1.0348, and (bottom) y=0.0024x+1.00 (due to weak correlation, the intercept in this last case only is set to a CF of unity).

Correction factors for AP–PA TBI and bilateral‐opposed TBI are displayed in 1, 2, respectively. In these figures the dashed lines represent 2% deviation from the linear fit, as well as the percentage deviation from the linear fit that includes 95% of the data points for the plot, as labeled. For Fig. 1, the percentage of data points within 2% of the linear fit is 58%/79%/100% for maximum/midpoint/mean lung dose, while the percentage deviation from the linear fit that includes 95% of the data is ±4%/3.25%/2% for the same plots. Similarly for Fig. 2, the percentage of data points within 2% of the linear fit is 25%/67%/50% for maximum/midpoint/mean lung dose, while the percentage deviation from the linear fit that includes 95% of the data is ±9%/4.5%/6% for the same plots. It should be noted that the correlation between bilateral thorax separation and lung dose correction factor is weakest in the mean lung dose case. This is due to the highly variable lung dose in the anterior portion of the lung (if no bolus or other blocking is used where arm tissue is absent). Specifically, bilateral thorax separation is not a good indication of how much anterior lung receives poor blocking from the patient's arms. For this plot only (i.e., Fig. 2, bottom) the intercept is set to a correction factor of unity: 95% of data points fall within ±6% of this line.

From Fig. 1, the relationship between AP–PA lung dose correction factor and thorax separation is highly dependent on which lung dose parameter is used to calculate the correction factors. The Task Group 29 protocol used dose calculated at the “middle of the lung” to calculate lung dose correction factors versus thorax separation for a ^60^Co beam, and analytically extended these results to higher photon energies. In that report, 80% of the correction factors fell within 1.5% of the linear fit for ^60^Co. The 3D planning results of the current study, for this sample of patients, indicate that the linear fit for lung maximum dose correction factors in the AP–PA case most closely resembles the calculated data presented for the 6 MV beam in Task Group 29. The 6 MV correction factors from Table 6 in TG‐29 are indicated by the triangular markers of Fig. 1 (top), with a connecting line for visualization.

From column five of Table 2 and from Fig. 2, the relationship between bilateral correction factors and patient separation is quite variable, perhaps even more so than the results reported by Van Dyk et al. for AP–PA TBI. This variability is due to the combined effects of treating through the patient's arms (where total bilateral separation is not necessarily indicative of how much arm “shielding” is present in each individual case) and overall highly inhomogeneous beam path, as compared to the AP–PA technique. Overall, the bilateral correction factors for mean lung dose are an average of 5% higher than the respective AP–PA correction factors for the same patients, but with high variability (i.e., standard deviation of 4%, column 6 of Table 2). In short, these data present two options for approximating a lung dose correction factor based on bilateral thorax separation in the absence of 3D dose calculation methods: a) use the midpoint dose or mean dose correction factor equations presented in Fig. 2, recognizing that the 95% confidence interval for these equations is ±4.5% and ±6%, respectively; or b) increase the correction factor calculated for AP–PA lung dose (based on AP–PA separation) by 5% for mean lung dose (standard deviation, 4%) to produce bilateral lung correction factors.

It should be noted that the calculations used in this study do not take into consideration lung shielding techniques (with associated thorax boosts, if necessary). Metal block shields or bag compensators (rice, saline, etc.) are often used in both AP–PA and bilateral TBI techniques in order to reduce lung dose to levels required by prescription and/or protocol. The analysis presented above confirms that decisions about the use and design of lung shielding based solely upon simple (i.e., non‐3D, homogeneous medium) calculation methods may undershield the lungs (or portions of the lungs), while subsequent thorax boosts may overdose the bony anatomy in the central chest. Three‐dimensional (3D) planning with inhomogeneity corrections gives a more accurate picture of doses received by the lungs and midline thorax, thereby improving the accuracy of shielding and boost decisions. In the absence of 3D planning techniques, the data presented in this work allow approximation of lung dose and midline thorax dose corrections.

### B. High‐energy TBI

**Figure 3:**
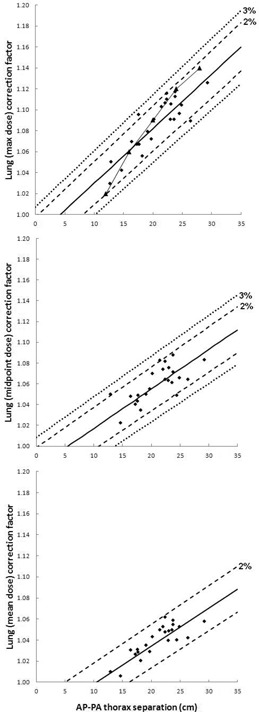
23 MV AP‐PA TBI lung dose correction factors. 23 MV AP‐PA thorax separation vs. lung correction factor, with linear fit, as calculated using lung maximum dose (top), lung midpoint dose (middle), and lung mean dose (bottom) for all 24 patients. Dashed trendlines indicate ±2% from the linear fit, as well as the percent error (i.e., ±%) for which 95% of the data points are included. Allowing x to represent AP‐PA separation and y to represent lung dose correction factor, the linear trendlines are described as follows: (top) y=0.0052x+0.9786, (middle) y=0.0038x+0.9791, and (bottom) y=0.0036x+0.9623. Triangular markers (with line for visualization purposes) indicate correction factors for 25 MV from Table 6 in TG‐29.^(2)^

**Figure 4:**
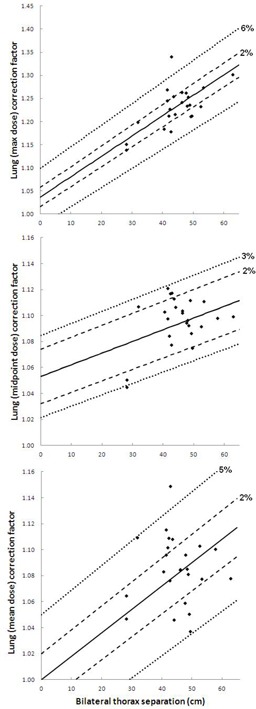
23 MV bilateral TBI lung dose correction factors. 23 MV bilateral thorax separation vs. lung correction factor, with linear fit, as calculated using lung maximum dose (top), lung midpoint dose (middle), and lung mean dose (bottom) for all 24 patients. Dashed trendlines indicate ±2% from the linear fit, as well as the percent error (i.e., ±%) for which 95% of the data points are included. Allowing x to represent bilateral separation and y to represent lung dose correction factor, the linear trendlines are described as follows: (top) y=0.0044x+1.0361, (middle) y=0.0009x+1.0531, and (bottom) y=0.0018x+1.00 (due to weak correlation, the intercept in this last case only is set to a CF of unity).

Using the 23 MV photon beam, planned dose to the umbilicus level was again not greatly impacted by inhomogeneity corrections. For the bilateral technique, using the monitor units calculated with ${\text{TDC}}_{\text{off}}$ and recalculating with applied inhomogeneity corrections, dose delivered to the midline umbilicus was 99.4% of the prescribed dose (on average) with a standard deviation of 1.7%. The same analysis with the AP–PA technique yields a dose delivered to the midline umbilicus of 99.8% (average) and standard deviation of 1.3%. However, dose distributions in the thorax are greatly impacted by tissue density correction: 3, 4, along with 3, 4, present the results for high‐energy (23 MV) TBI using the same methods as in the previous section. The choice of high‐energy photons requires fewer MU to deliver the same dose to the prescribed depth, and provides better dose homogeneity throughout the patient (excluding buildup effects, substantially noted in lower overall lung dose).^(2)^ The AP–PA lung dose correction factors displayed in Fig. 3 indicate that the percentage of data points within 2% of the linear fit is 92%/83%/100% for maximum/midpoint/mean lung dose, while the percentage deviation from the linear fit that includes 95% of the data is ±3%/3%/2% for the same plots. Meanwhile, for bilateral TBI with the higher energy photon beam, Fig. 4 shows that the percentage of data points within 2% of the linear fit is 67%/71%/54% for maximum/midpoint/mean lung dose, while the percentage deviation from the linear fit that includes 95% of the data is ±6%/3%/5% for the same plots.

**Table 3:** 23 MV TBI midline thorax dose results showing 23 MV bilateral vs. AP–PA TBI results for midline thorax dose, listed in order of increasing bilateral thorax separation

*# of Patients*	*Bilat. Thorax Separation (cm)*	*Bilat./AP MU Ratio*	*Bilat. Thorax Dose* $TDC_{on}/TDC_{off}$ *Ratio*	*AP Thorax Dose* $TDC_{on}/TDC_{off}$ *Ratio*
2	28	1.14	1.04	1.00
1	32	1.07	1.13	1.01
1	40	1.28	1.12	1.00
2	41	1.24	1.13	0.99
3	42	1.23	1.11	0.99
2	43	1.27	1.13	1.00
1	44	1.24	1.13	0.99
2	46	1.30	1.13	0.99
1	47	1.34	1.16	0.99
2	48	1.26	1.11	0.99
3	49	1.25	1.10	0.99
1	52	1.30	1.10	0.99
1	53	1.36	1.12	0.98
1	58	1.26	1.10	0.99
1	63	1.34	1.11	0.99
Mean		1.26	1.11	0.99
SD		0.08	0.03	0.01

**Table 4:** 23 MV TBI lung dose results showing 23 MV bilateral vs. AP–PA TBI results for lung dose, listed in order of increasing bilateral thorax separation. All ratios calculated from plans with TDC on, except the lung dose correction factors (CF). CF values represent the ratio of mean lung dose from fixed MU calculated with tissue density correction divided by the same lung dose parameter calculated without tissue density correction. Ratio of bilateral and AP–PA correction factors (column 6) is calculated for mean lung dose

*# of Patients*	*Bilat. Thorax Separation (cm)*	*Max Lung Dose Bilat./AP Ratio*	*Mean Lung Dose Bilat./AP Ratio*	*Mean Lung Dose Bilat./CF*	*Bilat/AP. CF Ratio*
2	28	1.06	1.01	1.06	1.05
1	32	1.02	0.99	1.11	1.04
1	40	1.08	1.03	1.08	1.05
2	41	1.13	1.05	1.11	1.06
3	42	1.05	0.99	1.10	1.07
2	43	1.14	1.05	1.13	1.07
1	44	1.09	0.99	1.05	1.00
2	46	1.11	1.00	1.08	1.05
1	47	1.18	1.05	1.06	1.01
2	48	1.06	0.96	1.09	1.06
3	49	1.02	0.93	1.06	1.01
1	52	1.07	1.01	1.10	1.05
1	53	1.06	0.93	1.08	1.03
1	58	1.10	0.93	1.10	1.06
1	63	1.05	0.93	1.08	1.02
Mean		1.08	0.99	1.08	1.04
SD		0.04	0.04	0.02	0.02

As is the case with the 6 MV data, the relationship between 23 MV bilateral lung dose correction factors and patient separation is highly dependent upon which lung dose parameter is used for calculation. The correlation between bilateral thorax separation and lung dose correction factor is weakest when the factors are calculated based upon mean lung dose. As a result, the intercept is set to a correction factor of unity for only the bottom plot in Fig. 4: 95% of data points fall within ±5% of this line. Similar also to the 6 MV results, the linear fit for AP–PA correction factors based on 23 MV maximum lung dose most closely resembles the calculated data presented in TG‐29: the 25 MV correction factors from Table 6 of TG‐29 are indicated by the triangular markers of Fig. 3 (top), with a connecting line for visualization.

## IV. CONCLUSIONS

In this study, a 3D treatment planning system was used to calculate lung dose distributions for TBI using AP–PA and bilateral opposed techniques at extended SSD. Dose distributions for each TBI delivery technique were examined both with, and without, tissue density (i.e., inhomogeneity) corrections. Dose calculations for bilateral TBI without inhomogeneity corrections underestimate dose by up to 20% in the thorax and up to 40% in the anterior lung. Lung dose corrections factors, similar to those presented in the AAPM Report 17 (Task Group 29), are calculated for both delivery techniques based on either AP–PA or bilateral thorax separation, and found to be highly dependent on the choice of lung dose parameter used to calculate the correction factors. Two methods are suggested for estimating bilateral TBI lung dose correction factors in the absence of more refined 3D calculation techniques, with associated uncertainties discussed in each case. Since the Task Group 29 data cannot be used to approximate bilateral lung dose correction factors, the correction methods suggested in this study, though simple and approximate, are better than not making any lung dose correction or incorrectly using AP–PA data to calculate corrections.
